# Intestine and brain TLR-4 modulation following *N*-acetyl-cysteine treatment in NEC rodent model

**DOI:** 10.1038/s41598-023-35019-5

**Published:** 2023-05-22

**Authors:** Ron Beloosesky, Ola Gutzeit, Yuval Ginsberg, Nizar Khatib, Michael G. Ross, Zeev Weiner, Osnat Zmora

**Affiliations:** 1 Department of Obstetrics and Gynecology, Rambam Health Care Campus, Haifa, Israel; 2 Ruth and Bruce Rappaport Faculty of Medicine, Israel Institute of Technology - Technion, Haifa, Israel; 3 Department of Obstetrics and Gynecology, Harbor-UCLA Medical Center, Torrance, CA USA; 4 Department of Pediatric Surgery, Shamir Medical Center, Be’er Ya’acov, Israel; 5 Sackler School of Medicine, Tel Aviv University, Tel Aviv, Israel

**Keywords:** Gastrointestinal diseases, Infant necrotizing enterocolitis, Neuroscience, Neuroimmunology

## Abstract

Necrotizing enterocolitis (NEC) brain injury is mediated through Toll-like receptor 4 (TLR4) on the intestinal epithelium and brain microglia. Our aim was to determine whether postnatal and/or prenatal NAC can modify NEC associated intestinal and brain TLR4 expression and brain glutathione levels in a rat model of NEC. Newborn Sprague–Dawley rats were randomized into three groups: Control (n = 33); NEC (n = 32)—hypoxia and formula feeding; and NEC-NAC (n = 34)—received NAC (300 mg/kg IP) in addition to NEC conditions. Two additional groups included pups of dams treated once daily with NAC (300 mg/kg IV) for the last 3 days of pregnancy: NAC-NEC (n = 33) or NAC-NEC-NAC (n = 36) with additional postnatal NAC. Pups were sacrificed on the fifth day, and ileum and brains harvested for TLR-4 and glutathione protein levels. Brain and ileum TLR-4 protein levels were significantly increased in NEC offspring as compared to control (brain 2.5 ± 0.6 vs. 0.88 ± 0.12 U and ileum 0.24 ± 0.04 vs. 0.09 ± 0.01, p < 0.05). When NAC was administered only to dams (NAC-NEC) a significant decrease in TLR-4 levels was demonstrated in both offspring brain (1.53 ± 0.41 vs. 2.5 ± 0.6 U, p < 0.05) and ileum (0.12 ± 0.03 vs. 0.24 ± 0.04 U, p < 0.05) as compared to NEC. The same pattern was demonstrated when NAC was administered only or postnatally. The decrease in brain and ileum glutathione levels observed in NEC offspring was reversed with all NAC treatment groups. NAC reverses the increase in ileum and brain TLR-4 levels and the decrease in brain and ileum glutathione levels associated with NEC in a rat model, and thus may protect from NEC associated brain injury.

## Introduction

Necrotizing enterocolitis (NEC) is a leading cause of premature infant morbidity and mortality^1^. In the USA, more than 3000 neonates are diagnosed with NEC annually^2^; 7% of infants weighing less than 1500 g are affected by NEC^3^ with mortality rate as high as 30%^4^.

NEC is both an infectious and an inflammatory disease that can affect various areas of the intestine, predominantly found in the region of the terminal ileum^5^. NEC etiology is considered multifactorial, a consequence of intestinal immaturity, microbial dysbiosis, and an exuberant inflammatory response.

Toll-like receptor 4 (TLR-4), which plays a critical role in the induction of protective host innate immune response, is an important factor in normal gut development^1^. Recent evidence suggest that TLR-4 is expressed at higher levels in the premature intestine and that it is more abundant in the intestine of infants with NEC^6,7^. The excessive inflammatory response observed in NEC is explained in part by over-expression and exaggerated activity of TLR-4 in the preterm intestine^6^.

The attachment of ligand to TLR-4 initiates a cascade of events that results in phosphorylation of kappa-B inhibitor in the cytosol and activation of NFKB P65, which translocates into the nucleus where it activates and regulates the transcription of genes related to inflammatory responses^8^.

In addition to intestinal injury in NEC, several studies reported significant severe neurodevelopmental disability among NEC survivors^9^. Recently it has been demonstrated in a NEC model that brain injury was associated with decreased brain glutathione levels^9^. However, the mechanisms associated with brain injury in NEC have not been fully elucidated.

*N*-acetyl cysteine (NAC) is a known anti-inflammatory and anti-oxidant agent used widely for paracetamol intoxication, and is safe to use during pregnancy (class B)^10,11^. Its anti-oxidant activity results from its conversion into metabolites that are capable of stimulating glutathione synthesis and promoting detoxification, and by its inherent function as a scavenger of free oxygen radicals.

TLR4 signaling plays a central role in the induction of NEC through the modulation of the epithelial cell barrier and regulation of the innate and adaptive immune responses. We hypothesized that modulation of TLR-4 effects could reduce the risk of NEC. In the present study, we sought to determine the protective effect of NAC on TLR-4 protein levels in neonatal intestine and brain, and to determine its effect on fetal brain glutathione levels in an established rat model of NEC.

## Methods

**Figure 1:**
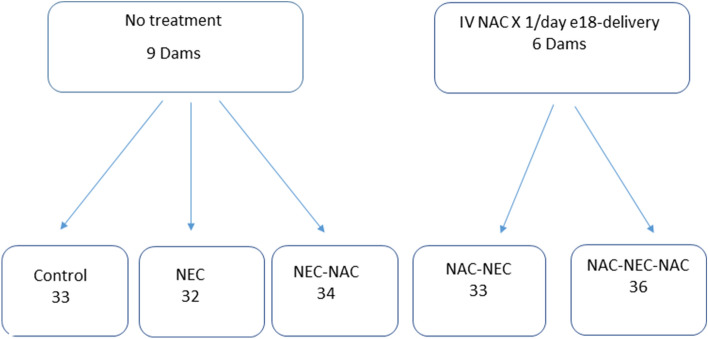
Flowchart of the different study groups. *IV* intravenous, *NAC* *N*-acetyl cysteine, *NEC* necrotizing enterocolitis, *IP* intraperitoneal, *NS* normal saline.

Pregnant Sprague–Dawley (SD) rats were obtained at day 11 of gestation and allowed to acclimate for 7 days before initiating the experiments (Fig. 1). The SD rats were maintained in light-controlled facilities with access to water and food ad libitum throughout the study, at ambient temperature (25 °C).

The first group of pregnant rats (nine dams) received no treatment during pregnancy and delivered spontaneously. After birth, the pups were divided into three groups.

- *Group 1* Control: 33 pups remained with their mothers (three dams) and were nursed at room temperature.

Sixty-six pups from six dams were separated from the dams and divided into Groups 2 and 3, as described below.

- *Group 2* NEC: 32 pups were transferred to an incubator (Ohio Medical Products, Madison, WI, USA), where they were fed thrice daily by gavage of 0.2 mL clean formula consisting of 15 g of Similac 60/40 (Abbott Nutritional, Columbus, OH, USA) in 75 mL of Esbilac canine milk replacement (PetAg Inc., Hampshire, IL, USA). The pups also received hypoxia exposure with 5% O_2_ and 95% N_2_ (for 10 min) and intraperitoneal injections of saline three times daily following each hypoxia exposure for 4 days.
- *Group 3* NEC-NAC: 34 pups were exposed to the same NEC conditions as pups in Group 2, with the addition of NAC treatment (*N*-acetyl cysteine, Martindale Pharma, Acetylcysteine 200 mg/ml, 2 g/10 ml ampoule reconstituted in water, 300 mg/kg intraperitoneal) instead of saline injections thrice daily, following each hypoxia exposure. We used the same compound that had been used for many years in our animal lab and in the hospital for clinical use in humans. We chose it in order to optimize the animal experiments to resemble human scenarios, such as an acetaminophen overdose. The dose of NAC we used is based on the recommendation of the Food and Drug Administration (FDA) for treating acetaminophen overdose. The FDA suggests that a safe and effective dose of NAC for treating acetaminophen overdose is between 150 mg/kg and 300 mg/kg per day^12^. Studies in perinatology have used doses ranging from 300 mg/kg^13^ to 1 g/kg^14^. In our current and previous research, we have utilized a dose of 300 mg/kg of NAC^15,16^. On a previous paper using an inflammatory model we used 600 mg/kg and 300 mg/kg and demonstrated that doses of 300 mg/kg were adequate for neuroprotection^17^.

To explore the possible protective effect of NAC during pregnancy, a second group of six pregnant rats received intravenous injections of NAC (300 mg/kg) once daily from gestational day 18 until delivery.

After birth, the pups were divided into Groups 4 and 5 as described below.

- *Group 4* NAC-NEC: 33 pups were separated from their dams and kept under the same conditions as Group 2 (NEC) above.
- *Group 5* NAC-NEC-NAC: 36 pups were separated from their dams and kept under the same conditions as Group 3 (NEC-NAC).

Pups in all study groups were euthanized on the fifth day of life.

### Sample collection

Pups were anesthetized with isoflurane and decapitated on their fifth day of life. Brains and the distal 3 cm of terminal ileum were harvested and immediately frozen in liquid nitrogen for further processing and analysis. Processing and analysis were performed on the mixture of intracellular and cellular components. Brain and terminal ileum TLR-4 protein levels were determined by Western blot, and terminal ileum and brain Glutathione levels determined by ELISA.

### Western blot analysis

Preparations of cell lysates and Western blot cells or tissues were lysed in RIPA buffer (phosphate-buffered saline containing 1% Nonidet P-40, 0.1% sodium dodecyl sulfate [SDS], 1 mmol/L Na3VO4, 4 mm phenylmethylsulfonyl fluoride and 0.05% [w/v] apro-tinin). Insoluble proteins were discarded by high-speed centrifugation at 2000 g for 10 min at 4 °C. Small volumes of lysate were taken to measure total protein concentration, using absorbance at 280 nm in triplicate by Nanodrop. Determination of the protein concentration samples was also compared with BSA standards, ensuring the standard was diluted in the same buffer as the samples, and a 50-μg sample was loaded into each well of a 12% SDS-PAGE acrylamide gel. After transferring and blocking to nitrocellulose membranes, the blots were incubated overnight with primary antibodies against the target protein, at 4 °C. The antibodies were diluted in blocking buffer according to the manufacturer’s recommendations.

Following incubation with primary antibodies and rinsing, the blots were incubated for 1 h at room temperature with HRP-conjugated secondary antibody, according to the manufacturer’s recommended ratio. Antibodies recognizing the TLR-4 (cat#NB100-56566 novus) were used in combination with a donkey anti-mouse horseradish peroxidase-conjugated secondary antibody (Jackson Immunoresearch Laboratories, West Grove, PA, USA). Following incubation with the diluted secondary antibody and washing with TBST the membrane was incubated with the peroxide solution according to the manufactory orders and enhanced to chemiluminescent (ECL) development solution (ab133406) by exposing to X-ray. Actin was used as a reference protein. Densitometric analysis was used to determine differences in protein expression.

### Densitometry analysis

To compare target protein expression levels among samples on the same blot or across blots, we normalized the bands by loading controls (housekeeping protein actin). To get an objective measure of the signal generated on Western blot, a densitometer was used to scan the blot or film, and imaging software was used to compare signals. The quantification reflects the ratio of each protein band relative to the control (actin). Films were subsequently imaged with ChemiDoc MP using the white light conversion screen and the silver stain (visible stain) application. The Band Analysis tools of IMAGELAB, software version 4.1 (Bio-Rad), were used to select and determine the back-ground-subtracted density of the bands in all the gels and blots.

### ELISA determinations

Offspring brain glutathione level was determined with a HT Glutathione Assay kit, catalog number: 7511-100-K (Bio-Techne Ltd., Minneapolis, MN, USA), according to the manufacturer’s instructions.

### Statistical analysis

Offspring brain and terminal ileum TLR-4 protein levels were compared for pups from the different groups (five brain samples and five terminal ileum samples from each group). Offspring brain glutathione protein levels were compared among pups from the different groups. All results were expressed as mean ± SD using one-way analysis of variance followed by post hoc tests for pairwise comparisons (Holm-Sidak method). Differences were significant at P < 0.05. Sigma Stat software, version 4.0 was used to perform the statistical analysis.

### Ethical approval

The protocols and procedures were approved by the Institutional Animal Care Committee at the Ruth and Bruce Rappaport Faculty of Medicine—Israel Institute of Technology (Technion) (Protocol number: IL001-01-2016). This study was carried out in strict accordance with the recommendations of the Israeli National Institute of Health guide for care and use of laboratory animals. Guidelines for the care and use of the animals approved by the local institution were also followed. All efforts were made to minimize animal suffering. The study was supervised by a veterinarian on a daily basis. The pain suffering of the rats was categorized as low.

## Results

### Clinical parameters

There were no differences in litter size, with a median of 11 (range 10–13) in NAC treated dams vs. 12.5 (range 10–14) in non-treated dams. Also, there were no differences in mean offspring birth weight between the two different groups of dams (6.7 + 0.7 gr with maternal treatment vs.6.5 + 0.8 in non-treated dams).

NEC was associated with pup mortality rate of 34% by the fifth day of life (11/32). When NAC treatment was administered to both dams and pups (NAC-NEC-NAC) pup mortality was significantly reduced to 11% (4/36). Mortality rate when NAC was administered to dams only (NAC-NEC) was 24% (8/33), and when NAC was administered only to offspring (NEC-NAC) was 26% (9/34). These were not significantly different from the NEC group. There were no mortalities in the control group.

### Brain and ileum TLR-4

**Figure 2:**
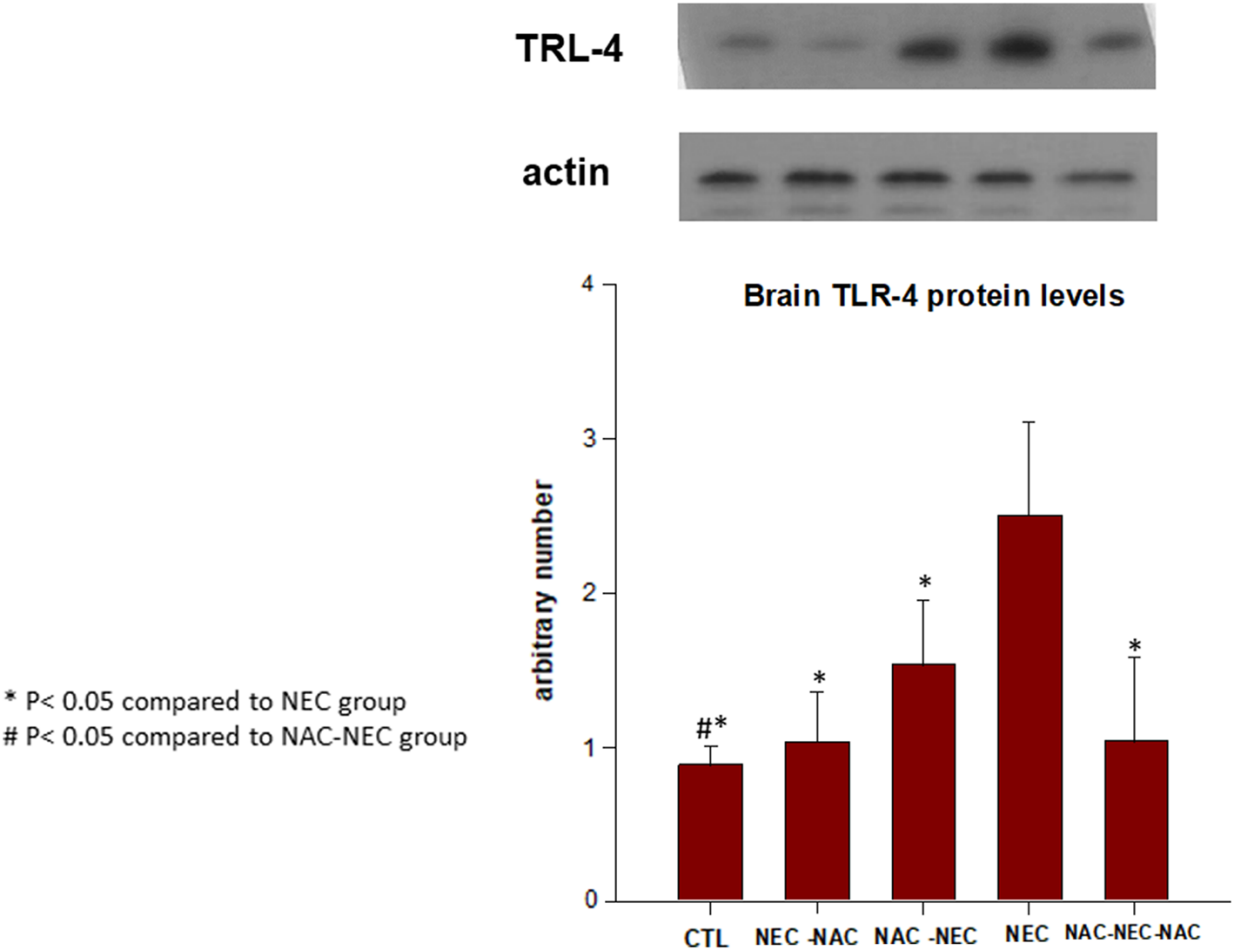
Offspring brain TLR-4 protein levels in Groups 1–5. NEC pups had significantly increased brain TLR-4 levels as compared with control pups; NAC to pregnant dams, offspring, or both dams and offspring significantly decreased offspring LLR-4 levels as compared with NEC pups (CTL, NEC, NEC-NAC, NAC-NEC and NAC-NEC-NAC). *Significant difference (P < 0.05) from NEC. #Significant difference (P < 0.05) from NAC-NEC. ^Significant difference (P < 0.05) from NEC-NAC. *NAC* *N*-acetyl cysteine, *NEC* necrotizing enterocolitis, *TLR-4* Toll-like receptor 4.

**Figure 3:**
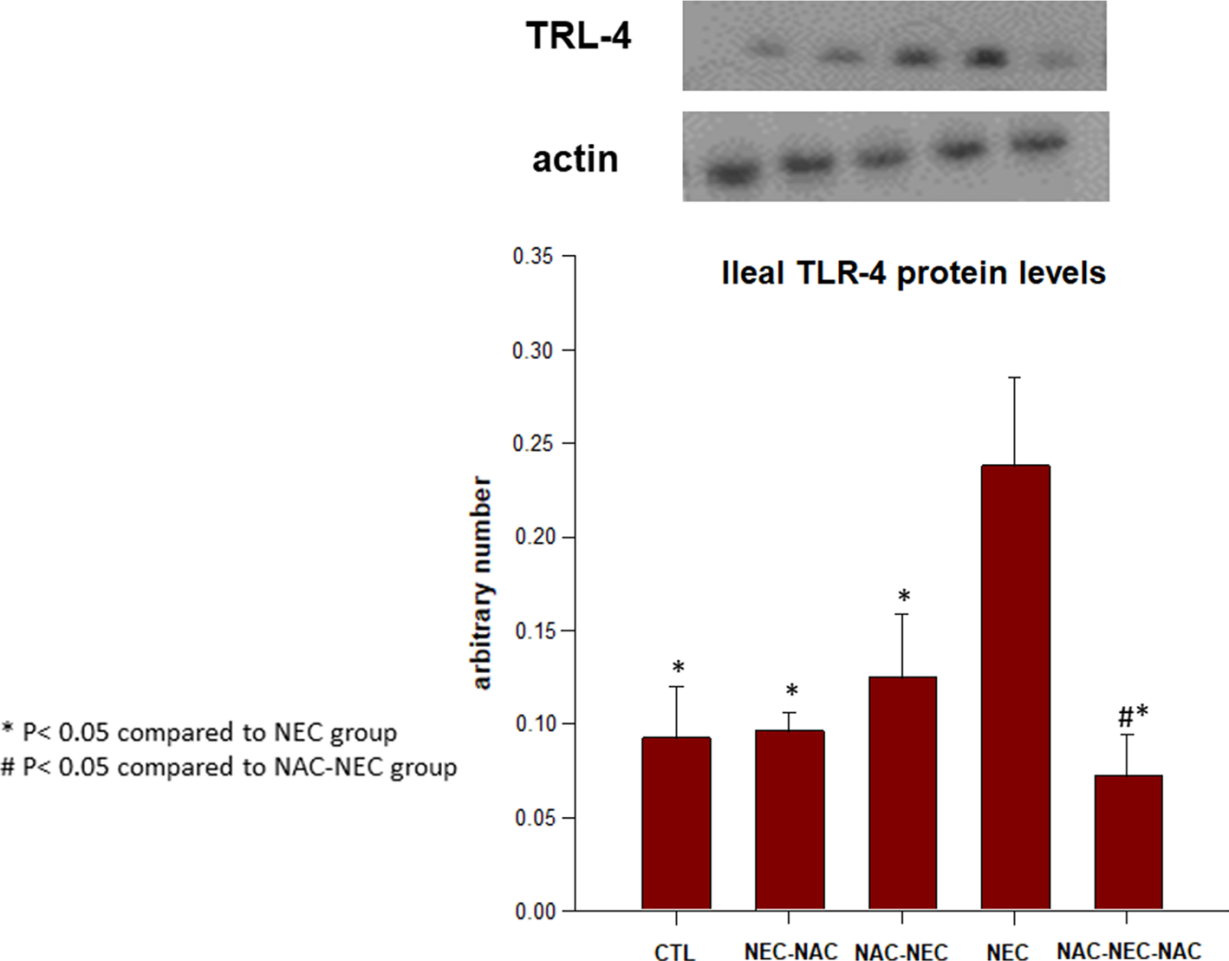
Offspring ileum TLR-4 protein levels in Groups 1–5. NEC pups had significantly increased ileal TLR-4 levels as compared with control pups; NAC to pregnant dams, offspring, or both dams and offspring significantly decreased offspring LLR-4 levels as compared with NEC pups (CTL, NEC, NEC-NAC, NAC-NEC and NAC-NEC-NAC). *Significant difference (P < .05) from NEC. ^#^Significant difference (P < 0.05) from NAC-NEC. *NAC* *N*-acetyl cysteine, *NEC* necrotizing enterocolitis, *TLR-4* Toll-like receptor 4.

TLR-4 protein levels in both brain and ileum were significantly increased in NEC offspring as compared to control (brain 2.5 ± 0.6 vs. 0.88 ± 0.12 U; ileum 0.24 ± 0.04 vs. 0.09 ± 0.02 U, respectively, p < 0.05). TLR-4 protein levels were significantly decreased when NAC was administered to pregnant dams only with subsequent NEC conditions (NAC-NEC) compared to both in the brain (1.53 ± 0.41 vs. 2.5 ± 0.6 U, p < 0.05) and ileum (0.12 ± 0.03 vs. 0.24 ± 0.04 U, p < 0.05). The same pattern of decreased brain and ileum TLR-4 protein levels compared to NEC was demonstrated when NAC was administered only to the offspring (NEC-NAC) (brain 1.03 ± 0.32 vs. 2.5 ± 0.6 U; ileum 0.09 ± 0.01 vs. 0.24 ± 0.04 U, p < 0.05) and to both dams and offspring (NAC-NEC-NAC) (brain 0.68 + 0.11 vs. 2.5 ± 0.6 U; p < 0.05; ileum 0.07 + 0.02 vs. 0.24 ± 0.04 U; p < 0.05) (Figs. 2 and 3).

### Brain glutathione level

**Figure 4:**
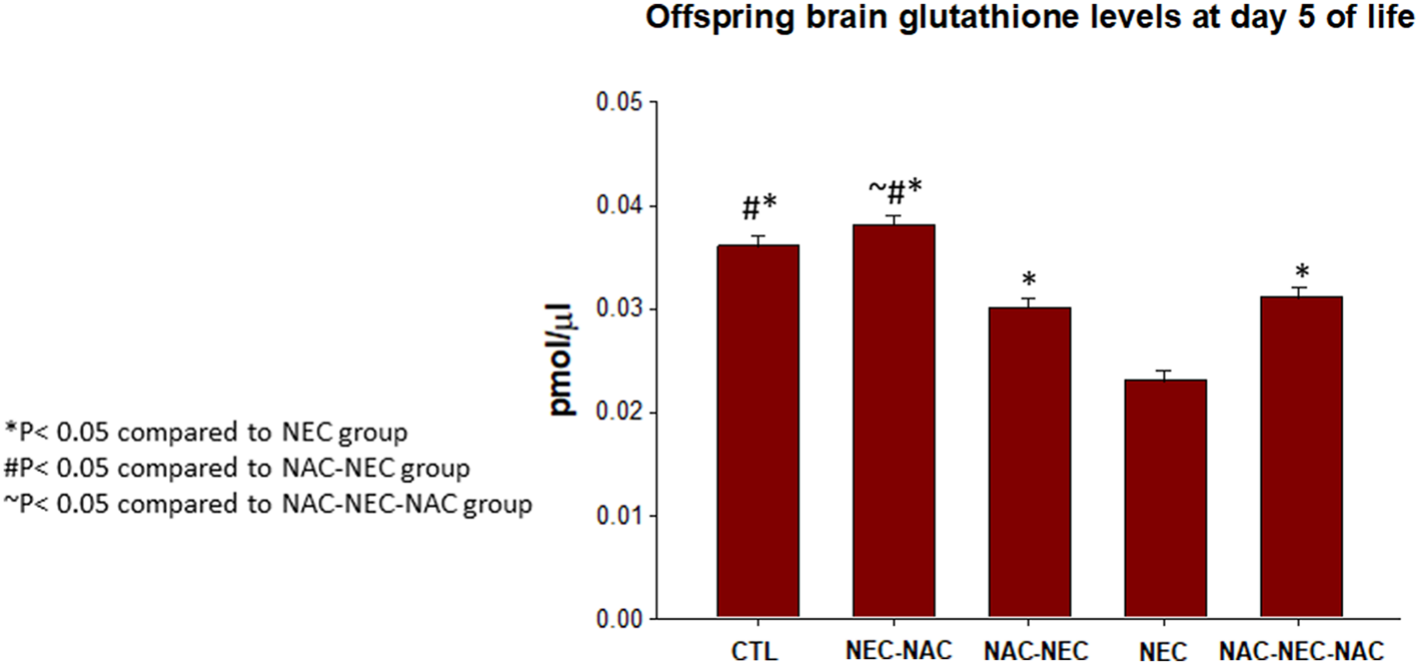
Offspring brain glutathione protein levels in Groups 1–5. NEC pups had significantly decreased brain glutathione levels as compared with control pups; NAC to pregnant dams, offspring, or both dams and offspring significantly increased offspring brain glutathione levels as compared with NEC pups. CTL, NEC, NEC-NAC, NAC-NEC and NAC-NEC-NAC. *Significant difference (P < 0.05) from NEC. ^#^Significant difference (P < 0.05) from NAC-NEC. ~ Significant difference (P < 0.05) from NAC-NEC-NAC. *NAC* *N*-acetyl cysteine, *NEC* necrotizing enterocolitis.

NEC offspring as compared to control offspring (0.023 ± 0.001 vs. 0.036 ± 0.001 pmol/ml, respectively, p < 0.05). Offspring brain glutathione levels were significantly increased when NAC was administered to pregnant dams only prior to offspring NEC conditions (NAC-NEC) as compared to NEC conditions alone (0.03 + 0.001vs. 0.023 ± 0.001 pmol/ml, respectively, p < 0.05). The same pattern was observed when NAC was administered only to offspring as compared to NEC (NEC-NAC; 0.038 + 0.001 vs. 0.203 ± 0.001 pmol/ml) or when NAC was administered to both dams and offspring compared to NEC (NAC-NEC-NAC; 0.031 + 0.001 vs. 0.203 ± 0.001 pmol/ml, respectively, p < 0.05) (Fig. 4).

### Iluem glutathione level

**Figure 5:**
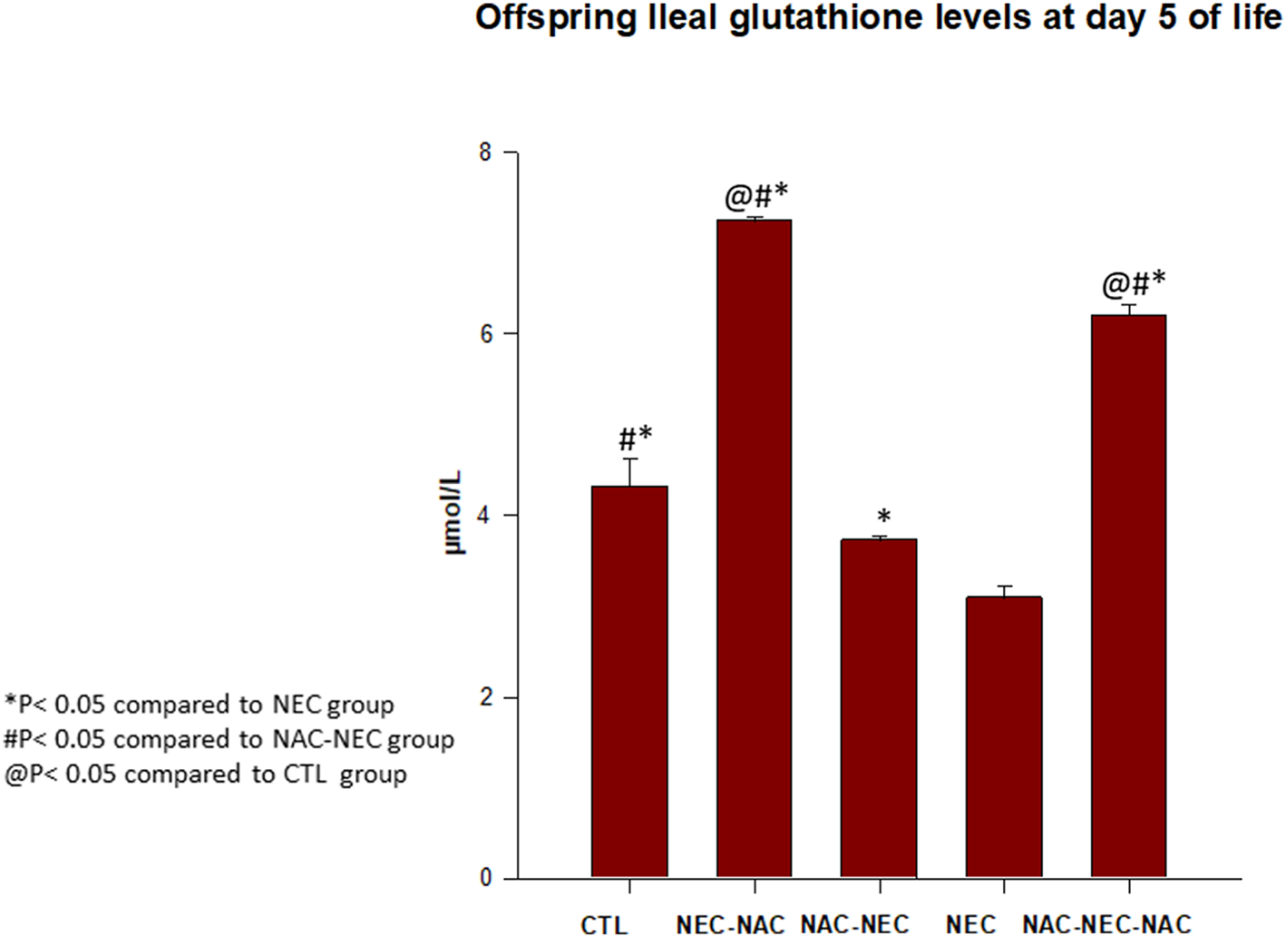
Offspring ileum glutathione protein levels in Groups 1–5. NEC pups had significantly decreased ileum glutathione levels as compared with control pups; NAC to pregnant dams, offspring, or both dams and offspring significantly increased offspring ileum glutathione levels as compared with NEC pups. CTL, NEC, NEC-NAC, NAC-NEC and NAC-NEC-NAC. *Significant difference (P < 0.05) from NEC. ^#^Significant difference (P < 0.05) from NAC-NEC. @Significant difference (P < 0.05) from CTL. *NAC* *N*-acetyl cysteine, *NEC* necrotizing enterocolitis.

Ileum glutathione levels were decreased in NEC offspring compared to control offspring (3.1 ± 0.11 vs 4.3 ± 0.3 µmol/l, respectively, p < 0.05). Offspring ileum glutathione were significantly increased when NAC was administered to pregnant dams only prior to offspring NEC (NAC-NEC) as compared to NEC (3.7 ± 0.04 vs 3.1 ± 0.11 µmol/l, respectively, p < 0.05). The same pattern was observed when NAC was administered only to offspring as compared to NEC (NEC-NAC 7.2 ± 0.04 vs 3.1 ± 0.11 µmol/l, respectively, p < 0.05, or when NAC was administered to both dams and offspring compared to NEC (NAC-NEC-NAC; 6.2 ± 0.11 vs 3.1 ± 0.11 µmol/l, respectively, p < 0.05) (Fig. 5).

## Discussion

In previous studies using this NEC model, we and others^18,19^ demonstrated features of NEC with severe bowel damage in pups, inflammatory changes, and increased oxidative stress accompanied by offspring brain injury. This model is based on insults that are recognized to contribute to the development of NEC in humans, including intestinal immaturity, hypoxia, and artificial hyperosmolar formula feeding. Dams are separated from the pups soon after birth to avoid breastfeeding, which is known to be protective against NEC.

In the current study, we have demonstrated that offspring with NEC had increased ileum TLR-4 protein levels as well as increased brain TLR-4 levels and decreased brain and ileum glutathione levels as compared to control. NAC administered to dams during pregnancy and/or to offspring under NEC conditions decreased offspring ileum and brain TLR-4 protein levels and increased brain and ileum glutathione levels as compared to NEC offspring.

In recent years exaggerated TLR-4 levels and increased activity in the immature intestine of preterm neonates has emerged as an inciting event in the pathophysiology of NEC. High TLR-4 activity in epithelial cells results in the initiation of an exaggerated immune response with increased production of pro-inflammatory cytokines and destruction of the mucosal barrier^1^. It has been demonstrated that TLR-4 contributes to bacterial translocation across the mucosal barrier, which facilitates the development of the severe illness observed during NEC^2^. The exact reason for increased expression of TLR-4 in enterocytes of infants with NEC has not been fully elucidated, though Soliman et al. suggested that platelet-activating factor was responsible for TLR-4 over-expression^20^.

Azjifurther demonstrated the importance of TLR-4 in the pathogenesis of NEC, showing that a specific deletion in the TLR4 locus in endothelial cells in a mouse model of NEC was associated with a significant reduction in NEC severity when compared to endothelial TLR-4 sufficient animals^21^.

Our novel finding that NAC administered to either dams or offspring decreased TLR-4 protein levels in offspring ileum is of great importance. These findings are consistent with reports of improved NEC features observed in previous studies following NAC treatment^22,23^.

We have recently demonstrated that NAC treatment was associated with decreased inflammatory response and attenuated activation of NFKB in a rodent NEC model. It is uncertain where NAC interferes in the cascade of the inflammatory response. Normally, following activation of TLR-4, a cascade of events occurs through the MyD88 dependent pathway which involves activation of the IRAK family of kinases, in which TAK1 activates the downstream kinase IKK in the final step, which in turn phosphorylates the NF-κB inhibitor IκBα, leading to ubiquitin-dependent IκBα degradation and NF-κB activation. This cascade results in transcription of inflammatory genes, including those encoding TNF-α, IL-1β, IL-6, IL-12p40, and cyclooxygenase^24^.

The findings of our current study might infer that the attenuation of the inflammatory response associated with NAC administration in NEC might be connected to modulation of the TLR-4 levels in the ileum and brain upstream of the cascade. A recent study by Niño et al. (2018) demonstrated a gut-brain signaling axis in a mouse model of NEC in which activation of intestinal TLR-4 signaling led to release of high-mobility group box 1 (HMGB1) protein in the intestine, which in turn, promoted activation of TLR-4 on brain microglial cells resulting in accumulation of reactive oxygen species, loss of oligodendrocyte premature cells, dysmyelination, and cognitive impairment. The authors further demonstrated the role of TLR-4 in mediating brain injury by generating mice lacking TLR-4 in microglia. The mice lacking microglial TLR-4 were protected from NEC associated brain injury. Further evidence for this intestinal-brain axis was generated by demonstration of reduced microglial activation with administration of intranasal anti-HMGB1 antibody to wild-type mice under NEC conditions^9^.

In the present study, we have demonstrated that under NEC conditions there is a significant increase in TLR-4 protein levels in the offspring brain. Our findings demonstrating reversal of NEC associated increase in intestinal and brain TLR-4 levels may explain the mechanism of NAC protective effect in both ileum and brain. Despite the data presented by Niño^9^ showing that NAC administration reduced microglial activation, they did not present any data on brain or gut TLR-4 levels in response to the treatment. Our data may explain the mechanisms behind the protective effects of NAC in both the ileum and brain.

We further demonstrated that the decrease in brain TLR-4 following NAC administration was associated with an increase in brain antioxidant glutathione levels, potentially enabling the brain to cope with increased oxidative stress and thus to amelioate brain injury. This finding is in accordance with the findings by Niño et al.^9^. Although we did not evaluate behavior in the current study, we demonstrated in a previous study on a hypoxia model (not NEC model), that NAC treatment significantly attenuated sensorimotor dysfunction in neonatal rats exposed to hypoxia^13^. In another study, we found that NAC protected the brain from injury as compared to LPS group, as demonstrated by MRI at 30 days of age^25^.

*N*-acetyl cysteine, a known anti-inflammatory and anti-oxidative agent, is considered safe for use during pregnancy (class B)^23^. Our data regarding litter size, offspring weight, and mortality support the safety of NAC administration. We did not analyze possible sex influences in our study as in previous research, despite finding differences in immunity parameters between the sexes and higher mortality in males, as we did not find any differences in gut structure, function, or NEC incidence between males and females^26^. NAC’s therapeutic properties stem from its action on the cystine-glutamate antiporter system and as an antioxidant to regulate the neuroinflammatory response^27,28^. Glutamate is implicated in fetal brain injury. Overexposure to glutamate and subsequent excess intracellular calcium influx, termed excitotoxicity, destroys neurons both in vivo and in vitro^29^. Thus, NAC may exert an additional therapeutic benefit through decreasing synaptic glutamate release, hence mitigating subsequent excitotoxic neurological damage^27^.

Interestingly, we have demonstrated that NAC was effective even when administered during pregnancy to dams long before exposing the offspring to NEC conditions. Our novel finding that maternal NAC administered long before delivery may protect the offspring from NEC associated changes in both intestine and brain is supported by a human study which has demonstrated rapid transfer of NAC from the mother to the fetus through the placenta, with umbilical cord concentrations frequently exceeding maternal concentrations^30^. Buhimshi et al. demonstrated in a maternal inflammation model in mice that maternal inflammation resulted in oxidative stress associated with maternal and fetal liver glutathione (GSH) precursor depletion, while maternal NAC restored both maternal and fetal oxidative balance and increased liver GSH levels in both dams and fetuses. Maternal NAC administration may be of benefit for newborns at increased risk of NEC, as it attenuates the inflammatory and oxidative responses. These novel findings, if confirmed by future studies, imply that administrating NAC to mothers in very early preterm birth (< 30 weeks) before delivery, may prevent NEC and improve the outcome of the newborns^14^.

The strength of our study is that we used an established rat model of NEC which replicates the key clinical features of NEC. This allowed us to assess the brain and intestine and to study the mechanisms mediating the development of NEC and NEC- associated brain injury. There are some limitations to our study. It is an animal study with a relatively small sample size and little clinically correlated data. Also, we studied the offspring at 5 days of age which might be too early a stage in brain development. Further studies should be performed at later stages with larger numbers.

In conclusion, we have demonstrated that prenatal and postnatal NAC could prevent an increase in TLR-4 protein levels in both ileum and brain in an established NEC model, while increasing brain glutathione levels. This study extends our understanding of the mechanisms associated with NEC injury and its prevention, and thus may help develop new strategies to cope with offspring injury associated with NEC.

## Supplementary Information

Supplementary Information 1.

Supplementary Information 2.

## Data Availability

All data generated or analyzed during this study are included in this published article [and its Supplementary Information files].

## References

[1] Mihi B, Good M (2019). Impact of toll-like receptor 4 signaling in necrotizing enterocolitis: The state of the science. Clin. Perinatol..

[2] Neu J (1996). Necrotizing enterocolitis: The search for a unifying pathogenic theory leading to prevention. Pediatr. Clin. North Am..

[3] Hunter CJ, Upperman JS, Ford HR, Camerini V (2008). Understanding the; susceptibility of the premature infant to necrotizing enterocolitis (NEC). Pediatr. Res..

[4] Blakely ML (2005). NEC Subcommittee of the NICHD Neonatal Research Network. Postoperative outcomes of extremely low birth-weight infants with necrotizing enterocolitis or isolated intestinal perforation: A prospective cohort study by the NICHD Neonatal Research Network. Ann. Surg..

[5] Balance WA, Dahms BB, Shenker N, Kliegman RM (1990). Pathology of neonatal necrotizing enterocolitis: A ten-year experience. J. Pediatr..

[6] Nanthakumar N (2011). The mechanism of excessive intestinal inflammation in necrotizing enterocolitis: An immature innate immune response. PLoS ONE.

[7] Gomart A, Vallée A, Lecarpentier Y (2021). Necrotizing enterocolitis: LPS/TLR4-induced crosstalk between canonical TGF-β/Wnt/β-catenin pathways and PPARγ. Front. Pediatr..

[8] Anderson KV (2000). Toll signaling pathways in the innate immune response. Curr. Opin. Immunol..

[9] Niño DF (2018). Cognitive impairments induced by necrotizing enterocolitis can be prevented by inhibiting microglial activation in mouse brain. Sci. Transl. Med..

[10] Riggs BS, Bronstein AC, Kulig K, Archer PG, Rumack BH (1989). Acute acetaminophen overdose during pregnancy. Obstet. Gynecol..

[11] Beloosesky R (2006). N-acetyl-cysteine suppresses amniotic fluid and placenta inflammatory cytokine responses to lipopolysaccharide in rats. Am. J. Obstet. Gynecol..

[12] Hendrickson RG (2019). What is the most appropriate dose of *N*-acetylcysteine after massive acetaminophen overdose?. Clin. Toxicol..

[13] Gutziet O (2021). Maternal N-acetyl-cysteine prevents neonatal hypoxia-induced brain injury in a rat model. Int. J. Mol. Sci..

[14] Buhimschi IA, Buhimschi CS, Weiner CP (2003). Protective effect of N-acetylcysteine against fetal death and preterm labor induced by maternal inflammation. Am. J. Obstet. Gynecol..

[15] Sharabi H (2019). Therapeutic N-acetyl-cysteine (Nac) following initiation of maternal inflammation attenuates long-term offspring cerebral injury, as evident in magnetic resonance imaging (MRI). Neuroscience.

[16] Beloosesky R (2009). Prophylactic maternal n-acetylcysteine before lipopolysaccharide suppresses fetal inflammatory cytokine responses. Am. J. Obstet. Gynecol..

[17] Beloosesky R, Weiner Z, Ginsberg Y, Ross MG (2012). Maternal N-acetyl-cysteine (NAC) protects the rat fetal brain from inflammatory cytokine responses to lipopolysaccharide (LPS). J. Matern. Fetal Neonatal Med.

[18] Kelly N (2004). The role of the glutathione antioxidant system in gut barrier failure in a rodent model of experimental necrotizing enterocolitis. Surgery.

[19] Sulistyo A, Rahman A, Biouss G, Antounians L, Zani A (2018). Animal models of necrotizing enterocolitis: Review of the literature and state of the art. Innov. Surg. Sci..

[20] Soliman A (2010). Platelet-activating factor induces TLR4 expression in intestinal epithelial cells: Implication for the pathogenesis of necrotizing enterocolitis. PLoS ONE.

[21] Azji I (2013). Endothelial TLR4 activation impairs intestinal microcirculatory perfusion in necrotizing enterocolitis via eNOS-NO-nitrite signaling. Proc. Natl. Acad. Sci. USA.

[22] Zmora O (2020). Prophylactic antenatal N-Acetyl Cysteine administration combined with postnatal administration can decrease mortality and injury markers associated with necrotizing enterocolitis in a rat model. PLoS ONE.

[23] Zmora O (2021). Maternal N-acetyl-cysteine prevents neonatal brain injury associated with necrotizing enterocolitis in a rat model. Acta Obstet. Gynecol. Scand..

[24] Liu T, Zhang L, Joo D, Sun SC (2017). NF-κB signaling in inflammation. Signal Transduct. Target Ther..

[25] Sharabi H (2019). Therapeutic N-acetyl-cysteine (Nac) following initiation of maternal inflammation attenuates long-term offspring cerebral injury, as evident in magnetic resonance imaging (MRI). Neuroscience.

[26] Baek O (2021). Sex-specific survival, growth, immunity and organ development in preterm pigs as models for immature newborns. Front Pediatr..

[27] Dean O, Giorlando F, Berk M (2011). N-acetylcysteine in psychiatry: Current therapeutic evidence and potential mechanisms of action. J. Psychiatry Neurosci..

[28] Durieux AM (2015). Targeting glia with N-acetylcysteine modulates brain glutamate and behaviors relevant to neurodevelopmental disorders in C57BL/6J mice. Front. Behav. Neurosci..

[29] Park E (2010). Correlation between extracellular glutamate release and neuronal cell death in an eleven vessel occlusion model in rat. Brain Res..

[30] Wiest DB (2014). Antenatal pharmacokinetics and placental transfer of N-acetylcysteine in chorioamnionitis for fetal neuroprotection. J. Pediatr..

