# Supplementary figures and images for: Intestine and brain TLR-4 modulation following N-acetyl-cysteine treatment in NEC rodent model
Source: Sci Rep. 2023 May 22;13:8241. doi: 10.1038/s41598-023-35019-5 (PMC10203358; doi:10.1038/s41598-023-35019-5)

## Slide 1
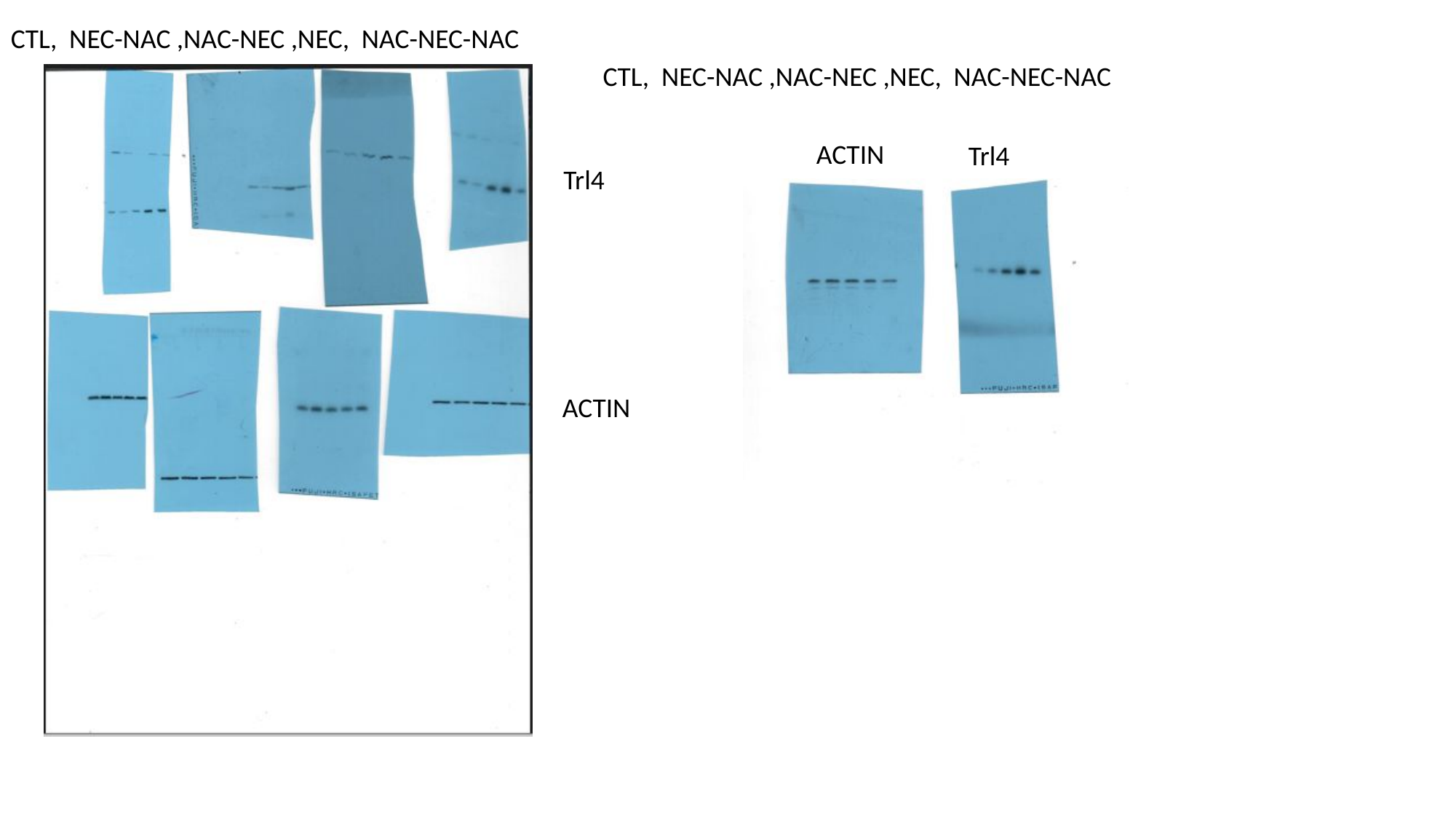

CTL, NEC-NAC ,NAC-NEC ,NEC, NAC-NEC-NAC
CTL, NEC-NAC ,NAC-NEC ,NEC, NAC-NEC-NAC
ACTIN
Trl4
Trl4
ACTIN

Supplement: Supplementary file 1 — Supplementary Information 1. [file 41598_2023_35019_MOESM1_ESM.pptx]

## Slide 1
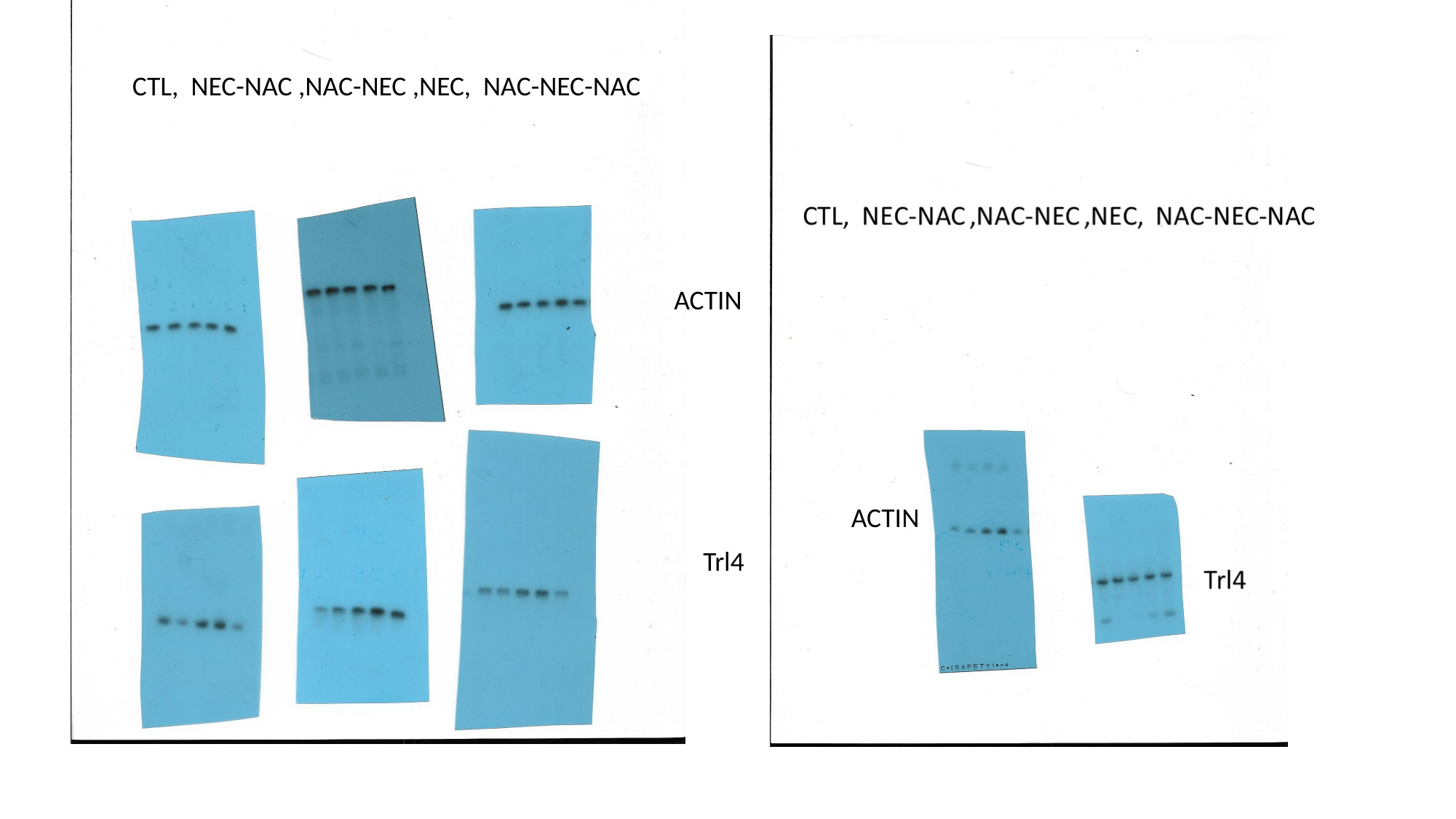

CTL, NEC-NAC ,NAC-NEC ,NEC, NAC-NEC-NAC
ACTIN
ACTIN
Trl4

Supplement: Supplementary file 2 — Supplementary Information 2. [file 41598_2023_35019_MOESM2_ESM.pptx]
